# Effects of suppression of phosphate transporter 4;4 on CO_2_ assimilation in rice

**DOI:** 10.1007/s10265-025-01638-4

**Published:** 2025-04-18

**Authors:** Ryosei Harada, Takaya Sugimoto, Yuki Takegahara-Tamakawa, Amane Makino, Yuji Suzuki

**Affiliations:** 1 Graduate School of Arts and Sciences, Iwate University, Morioka, Japan; 2 Faculty of Agriculture, Iwate University, 3-18-8 Ueda, Morioka, 020-8550 Japan; 3 Graduate School of Agricultural Science, Tohoku University, Sendai, Japan; 4 Present Address: Institute for Excellence in Higher Education, Tohoku University, Sendai, Japan

**Keywords:** CO_2_ assimilation, Inorganic phosphate, Phosphate transporter, Rice (*Oryza sativa*), RNAi suppression

## Abstract

Homeostasis of inorganic phosphate (P_i_) in the chloroplasts is essential for healthy CO_2_ assimilation. When P_i_ in chloroplasts is insufficient, the increase in the CO_2_ assimilation rate (*A*) with an increase in CO_2_ level is restricted, whereas *A* per unit total protein level moderately decreases under low-to-normal CO_2_ levels. Some phosphate transporters (PHT) are localized in the chloroplast envelope; however, their contribution to the maintenance of P_i_ homeostasis for CO_2_ assimilation has rarely been reported. In this study, we generated transgenic rice plants with RNAi-suppressed *PHT4;4*, one of the two genes of chloroplast envelope-localized PHT, and examined the changes in the characteristics of CO_2_ assimilation. In three transgenic lines, the mRNA levels of *PHT4;4* decreased by approximately 80% without a notable decrease in total leaf-P levels or total leaf-N levels, which is thought to approximately correspond to total protein levels. *A* in the transgenic plants tended to be slightly lower than that in the wild-type plants, irrespective of the CO_2_ level, and typically increased to saturation with increasing CO_2_ levels. *A* per unit total leaf-N level in transgenic plants tended to be slightly lower than that in wild-type plants. These results indicate that substantial *PHT4;4* suppression caused slight symptoms of P_i_-limited CO_2_ assimilation. Therefore, PHT4;4 is suggested to be involved in the maintenance of chloroplast P_i_ homeostasis for healthy CO_2_ assimilation, although its contribution is minor.

**Supplementary Information:**

The online version contains supplementary material available at 10.1007/s10265-025-01638-4.

## Introduction

CO_2_ assimilation occurs through the Calvin–Benson cycle using ATP and NADPH supplied by the light reactions (Calvin 1962; Heldt and Piechulla 2011). According to the biochemical models of C_3_ photosynthesis, the capacities of Rubisco carboxylation and regeneration of the Rubisco substrate ribulose 1,5-bisphosphate, determined by photosynthetic electron transport rates and NADPH supply, are assumed to limit the CO_2_ assimilation rate (*A*) (Farquhar et al. 1980; von Caemmerer 2000). Furthermore, the homeostasis of inorganic phosphate (P_i_) is essential for healthy CO_2_ assimilation because P_i_ availability for ATP synthesis in chloroplasts is also assumed to limit *A* (McClain and Sharkey 2019; Sharkey 1985a). P_i_-limited CO_2_ assimilation is evoked by substantially elevated CO_2_ levels and a combination of elevated CO_2_ levels and low O_2_ levels, low temperatures, or sink limitations under high irradiance (Busch and Sage 2017; Fabre et al. 2019; Sage and Sharkey 1987; Sharkey 1985b; Yang et al. 2016). In these cases, the increase in *A* with increasing CO_2_ levels is notably hindered, leading to the CO_2_ insensitivity of *A* and even its reverse CO_2_ sensitivity.

Currently, atmospheric CO_2_ levels continue to rise. Elevated CO_2_ levels have been shown to decrease P levels in plant tissue including leaves (Loladze 2014). However, P_i_ limitation in CO_2_ assimilation is not primarily caused by P nutrition deficiency but by an imbalance in photosynthetic carbon and phosphate metabolism. P_i_ for chloroplast ATP synthesis is regenerated via the utilization of the Calvin–Benson cycle metabolite triose phosphate for sucrose synthesis in the cytosol and starch synthesis in the chloroplasts. During sucrose synthesis, the transport of triose phosphate to the cytosol is accompanied by the countertransport of P_i_ to the chloroplast, which is carried out by the triose phosphate/phosphate translocator (TPT) localized in the chloroplast envelope (Flügge and Heldt 1984; Riesmeier et al. 1993). In the above-mentioned cases of P_i_-limited CO_2_ assimilation, the activity of CO_2_ assimilation surpasses that of sucrose and starch synthesis, leading to a shortage of P_i_ transported from the cytosol to the chloroplast and regenerated within the chloroplasts for chloroplastic ATP synthesis (McClain and Sharkey 2019; Sharkey 1985a). Suppression or defects in enzymes involved in sucrose or starch synthesis cause symptoms of P_i_-limited CO_2_ assimilation (Micallef et al. 1996; Strand et al. 2000; Yang et al. 2016), whereas suppression of the Calvin–Benson cycle enzyme triose phosphate isomerase causes similar symptoms (Suzuki et al. 2022a).

TPT is considered a major P_i_ supplier to chloroplasts, as it is estimated to account for 20–30% of chloroplastic P_i_ uptake using isolated chloroplasts of *TPT*-antisense potato plants, with its mRNA barely detectable (Riesmeier et al. 1993). This value is comparable to the estimated percentage of fixed C exported through the TPT for sucrose synthesis (McClain and Sharkey 2019). Transgenic plants or mutants with decreased or defective TPT activity show symptoms of P_i_-limited CO_2_, that is, suppression of *A* under elevated CO_2_ levels and diminished CO_2_ sensitivity of *A* (Lee et al. 2014; Riesmeier et al. 1993; Schneider et al. 2002; Walters et al. 2004; Yang et al. 2016). In addition, *TPT* suppression affects *A* under low-to-normal CO_2_ levels and high irradiance. For example, in *TPT*-knockdown Arabidopsis mutants, Rubisco carboxylation capacity *V*_cmax_, which was deduced from the initial slope of the CO_2_ response curve of *A*, was smaller than that in wild-type plants by approximately 25–50% under low to high temperatures, without differences in total soluble protein levels from wild-type plants (Yang et al. 2016). In these mutants, the CO_2_ sensitivity of *A* did not diminish under normal or high temperatures (Yang et al. 2016). These observations show that P_i_ limitation suppresses *A* at low-to-normal CO_2_ levels without diminishing the CO_2_ sensitivity of *A*, although the underlying mechanisms remain unknown. A 20% reduction in *A* per unit total protein level under normal CO_2_ levels has also been observed in *TPT*-antisense tobacco plants, with a reduced TPT activity by 70% (Häusler et al. 2000a).

In addition to TPT, some phosphate transporters (PHT) are involved in P_i_ transport from the cytosol to the stroma. Plant PHTs which comprise various molecular species, including the PHT1–6 families, are expressed in various plant tissues and are responsible for various roles, such as root P_i_ uptake and internal P_i_ transport (Fabiańska et al. 2019; Versaw and Garcia 2017; Wang et al. 2018). Certain members of the PHT2 and PHT4 families are localized in the chloroplast envelope. Contribution of these PHTs to chloroplastic P status has been examined, although the measurement conditions might not be favorable for photosynthesis. Defect and overexpression of PHT2;1, a putative low-affinity type transporter, notably decreased and increased, respectively, the total P content of isolated chloroplasts in rice (Bouain et al. 2022). Defect of Arabidopsis PHT4;4, which was also shown to have transport activity for P_i_ and ascorbate (Miyaji et al. 2015), scarcely affect *in-vivo* stromal P_i_ concentration measured using Förster resonance energy transfer-(FRET) based P_i_ sensor, whereas defect of TPT notably decreased *in-vivo* stromal P_i_ concentration (Raju et al. 2024). Changes in *A* or the behavior of the photosystems were also examined. Defect of PHT2;1 decreased *A* under normal CO_2_ levels by approximately 25% in rice (Liu et al. 2020). Defect of PHT4;4 diminished NPQ, accompanied by decreased stromal ascorbate levels and suppressed xanthophyll cycle operations in Arabidopsis (Miyaji et al. 2015). Recently, knockout of the gene of CrPHT4-7 in *Chlamydomonas reinhardtii*, which exhibited notable similarity to Arabidopsis PHT4;4, reduced ATP levels and diminished NPQ, leading to retarded growth, particularly under high irradiance (Tóth et al. 2024), although CrPHT4-7 did not show notable ascorbate transport activity. Despite these pieces of information on chloroplastic P status and photosynthesis, it has not been reported whether these mutants showed the symptoms for P_i_-limited CO_2_ assimilation. Therefore, whether chloroplast envelope-localized PHTs are crucial in the maintenance of chloroplastic P_i_ homeostasis for healthy CO_2_ assimilation remains unclear.

In rice, PHT4;4, a homolog of Arabidopsis PHT4;4, is localized in the chloroplast envelope, is relatively highly expressed in leaves, and exhibits P_i_ transport activity, as demonstrated by a complementation test using yeast defects in PHT (Li et al. 2020). Therefore, rice PHT4;4 may play a role in maintaining chloroplast P_i_ homeostasis for CO_2_ assimilation. To validate this hypothesis, the gene expression of *PHT4;4* was suppressed using RNA interference (RNAi) in rice plants. Changes in the mRNA levels of *PHT4;4* were examined in mature leaves, where the mRNA levels were maximal during leaf development. Total leaf-P levels were determined to evaluate whether PHT4;4 is involved in P_i_ supply to leaves. *A* was measured under high irradiance and different CO_2_ levels to examine whether the CO_2_ insensitivity of *A* was enhanced in transgenic plants. Considering that the total leaf-N level is a primary determinant of *A* (Evans 1989; Makino et al. 1988, 1994; Seemann et al. 1987) and approximately corresponds to the total protein level, *A* per unit total leaf-N level (*A*/N) was also examined to assess the symptoms of P_i_-limited CO_2_ assimilation.

## Materials and methods

### Generation of transgenic plants

The gene expression *PHT4;4* was suppressed by the RNA interference (RNAi) technique using the binary vector pIPKb027 (Himmelbach et al. 2007) in rice (*Oryza sativa* L.), as described by Suzuki et al. (2022b). Briefly, a DNA fragment of 250 bp containing the 3′-end of the coding region and the 3′-untranslated region of the transcript of *Os09 t0570400-1* [Rice Genome Annotation Project Database (RAP-DB; Kawahara et al. 2013)] was amplified by reverse-transcription PCR (RT-PCR) using total RNA extracted from leaf blades of rice (‘Notohikari’). The amplified DNA fragment was introduced into the pIPKb027 vector to generate an RNAi vector. The primer pair used was 5′-CACCGTTCTTGAATGAGCTGTAGATG-3′ and 5′-GTAAGGTAATTCACTGGCTCC-3′. The vector was introduced into rice (‘Notohikari’) using the *Agrobacterium* transformation method (Toki et al. 2006). The T_0_ progenies of transgenic plants were grown hydroponically in an environmentally controlled growth chamber, as described later. Environmental conditions were identical to those used after seedling transplantation. Transgenic lines with decreased *PHT4;4* mRNA levels were selected using semi-quantitative RT-PCR, as described later. Lines 2, 7, and 11 were allowed to self-fertilize, and the T_1_ seeds were collected.

### Plant culture and sampling

Wild-type rice plants (‘Notohikari’) and T_1_ progenies of the transgenic plants were grown hydroponically in an environmentally controlled growth chamber (NC-411HC, NKsystem, Osaka, Japan) as previously described (Suzuki et al. 2021) with slight modifications. Seedlings were grown in tap water adjusted to a pH of 5.5 for 21 days after sowing. The maximum light intensity during the daytime was a photosynthetic photon flux density (PPFD) of 500 µmol quanta m^–2^ s^–1^ with a photoperiod of 14 h. The maximum day and night temperatures were 24 and 19 °C, respectively. The CO_2_ levels and relative humidity were adjusted to 40 Pa and 60%, respectively. Before transplantation, homozygotes were selected using the comparative cycle threshold method (Prior et al. 2006), as described by Ogawa et al. (2012). The cytoplasmic glutathione reductase gene (*GR*; *Os02 g0813500*) and spacer region between the triggers in the RNAi vector were used to detect the endogenous reference gene and transgene, respectively. The primer pair and probe for *GR* were described by Ogawa et al. (2012). The primer pair and probe for the transgene were 5′-TCTTTTTCAGGACTCGGAAGCT-3′ and 5′-TTTTCTATGCACTAACTATTCATCATGTG-3′ and 5′-TTTGGCTTATTTAGTTTTTTGGG-3′, respectively. Wild-type and homozygous seedlings were transplanted into hydroponic culture in the same growth chamber. The maximum light intensity during the daytime and the maximum day and night temperatures were changed to a PPFD of 1,000 µmol quanta m^–2^ s^–1^ and 27 and 22 °C, respectively. The nutrient solution described by Makino et al. (1988) was used. Nutrient solutions were renewed once per week. The strength of the nutrient solution was changed according to plant growth as follows: 1/3 strength for 2 weeks, 1/2 strength for 1 week, 2/3 strength for 1 week, full strength for 1 week, and full strength with increasing N concentration to 6 mM (3 mM NH_4_NO_3_). Two weeks after the start of 6 mM N feeding, the uppermost and fully expanded leaves were used for CO_2_ gas exchange measurements. After the measurements, the leaves were collected, and their fresh weights and areas were measured. A scanner and ImageJ (version 1.53k; Schneider et al. 2012) were used to measure the leaf area. The leaves were frozen in liquid nitrogen and stored at –80 ºC until use. The uppermost and fully expanded leaves were also collected for RNA analysis according to the methods of Suzuki et al. (2007). For wild-type plants, leaves that emerged from their sheaths by 50%, uppermost and fully expanded leaves, and penultimate and fully expanded leaves were also collected to examine changes in the mRNA levels of *PHT4;4* depending on leaf age.

### Measurements of gas exchange

*A* was measured using a portable gas exchange system (LI-6400XT; Li-Cor, Lincoln, NE, USA). Plants were placed in an environmentally controlled growth chamber (NC-411HC, NK system, Osaka, Japan) operated at a PPFD of 300 µmol quanta m^−2^ s^−1^, a CO_2_ level of 40 Pa, and an air temperature of 27 °C for at least 30 min. After the plants were taken out from the growth chamber, measurement of *A* was immediately initiated at a PPFD of 1,500 µmol quanta m^−2^ s^−1^, an ambient CO_2_ partial pressure (*pCa*) of approximately 16 Pa, leaf temperature of 25 °C, and a leaf-to-air vapor pressure difference of 1.0–1.2 kPa. After *A* increased and reached a steady state, the CO_2_ level was adjusted to obtain *pCa* values of 24, 40, 60, 80, and 120 Pa.

### Biochemical assays

Frozen leaves were homogenized using a pestle in a chilled mortar containing 50 mM sodium citrate buffer (pH 7.5), containing 2 mM iodoacetic acid, 0.8% (v/v) 2-mercaptoethanol, and 5% (v/v) glycerol. An aliquot was subjected to Kjeldahl digestion (Makino et al. 1984), followed by total N determination using Nessler’s reagent and total P determination using a vanadate-molybdate reagent. SDS-treated samples for the determination of Rubisco were prepared as described by Suzuki et al. (2022c) and kept at – 30 °C until use. The amount of Rubisco was determined by formamide extraction of Coomassie Brilliant Blue R-250-stained bands corresponding to the large and small subunits of Rubisco separated by SDS-PAGE as described by Makino et al. (1985) and Suzuki et al. (2022c).

### RNA analysis

Total RNA was extracted as described by Suzuki et al. (2004), with slight modifications (Suzuki et al. 2009a). Semi-quantitative RT-PCR analysis of the mRNA levels of *PHT4;4*, *PHT2;1*, *TPT*, the genes of the α subunit of chloroplast ATP synthase (*ATPA*), the cytochrome *f* subunit of cytochrome *b*_6_/*f* complex (*PETA*), and Rubisco large and small subunits (*RBCL* and *RBCS*, respectively), and *18 S rRNA* levels was performed as described by Suzuki et al. (2022a). THUNDERBIRD^®^ Next SYBR^®^ qPCR Mix (TOYOBO) was used as the quantitative PCR reagent. The mRNA levels were divided by *18 S rRNA* levels in each sample and converted to relative levels. Primer pairs for *PETA*, *RBCL*, *RBCS*, and *18 S rRNA* were presented by Suzuki et al. (2022a). The other primer pairs used were as follows. 5′-GCATCTGCACCAAGAAGTGTCA-3′ and 5′-TGGGCAGGAGCTTGTTCAG-3′ for *PHT4;4*, 5′-ACAGCCCATTGCACTTCATAAA-3′ and 5′-AAACTTTTTGGAGTCAGCCTATCTCT-3′ for *PHT2;1*, 5′-TGAGCAATTACCAACAGCCAGTT-3′ and 5′-GTGGATTCTTCATCTAAGCTGTACAGA-3′ for *PHT4;1*, 5′-CAAGGCTAAGATCGAAGAGGAGAA-3′ and 5′-CCATATTCACAGAAACCACCTCATC-3′ for *TPT*, and 5′-TCCGCGAACGTATTGAACAAT-3′ and 5′-CCCATCCCCCACTTGAACT-3′ for *ATPA*. To determine the mRNA level of *RBCS*, a part of the highly homologous region among *RBCS2*, *3*, *4*, and *5*, which is highly expressed in rice leaf blades (Suzuki et al. 2009b), was amplified using semi-quantitative RT-PCR.

### Statistical analysis

Statistical analysis was conducted by analysis of variance followed by the Tukey–Kramer test or the Dunnett test using R (version 4.4.2; Ihaka and Gentleman 1996) and the package"multicomp"(version 1.4–26; Hothorn et al. 2008).

## Results

**Fig. 1:**
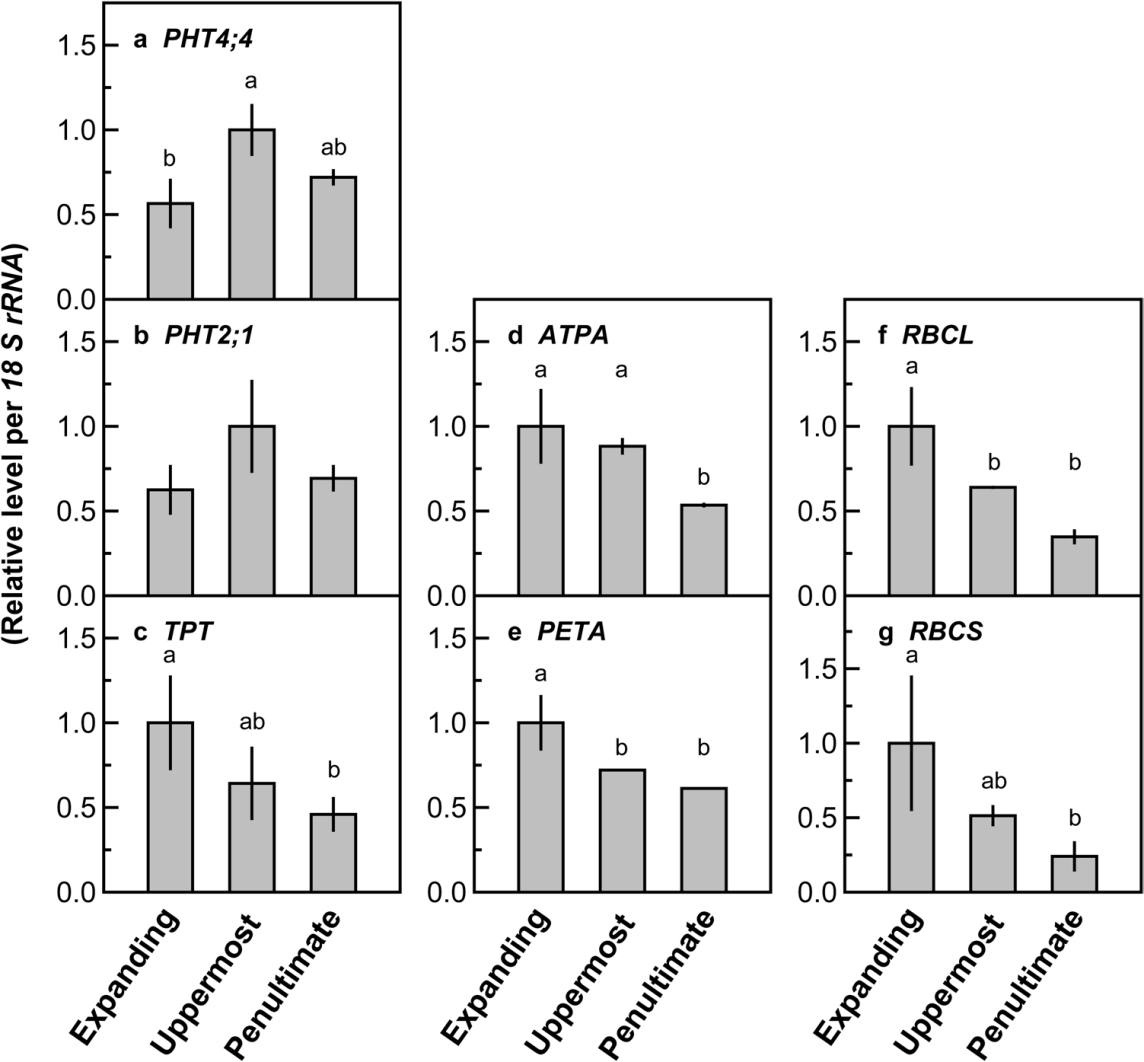
mRNA levels of the genes of phosphate transporters *PHT4;4* (**a**) and *PHT2;1* (**b**), triose phosphate/phosphate translocator (*TPT*) (**c**), the α subunit of chloroplast ATP synthase (*ATPA*) (**d**), the cytochrome *f* subunit of cytochrome *b*_6_/*f* complex (*PETA*) (**e**), and Rubisco genes *RBCL* (**f**) and *RBCS* (**g**) in leaves at different positions of wild-type rice plants. Expanding, Uppermost, and Penultimate represent expanding, uppermost and fully expanded, and penultimate and fully expanded leaves, respectively. The mRNA levels of each gene were divided by the *l8 S rRNA* levels, which were measured in the same samples and then converted to relative values. The maximal average value of mRNA level/*18 S rRNA* level among the leaf positions was defined as 1. Data are presented as means ± standard deviation (*n* = 3). Analysis of variance was performed on all leaves, followed by Tukey–Kramer’s test. Symbols with the same letter are not significantly different (*p* < 0.05)

The effects of RNAi suppression of *PHT4;4* on its mRNA levels were to be examined when the mRNA levels were maximal during leaf development. The mRNA levels of *PHT4;4* were reported to be higher in new leaves than in old leaves (Li et al. 2020). We also determined the mRNA levels of *PHT4;4* in wild-type plants using leaves at three different positions: expanding leaves, uppermost and fully expanded leaves, and penultimate and fully expanded leaves (Fig. 1). The expanding leaves corresponded to young leaves, in which the mRNA levels and activities of Calvin–Benson cycle enzymes and *A* rapidly increase (Makino et al. 1983; Yamaoka et al. 2016), whereas the uppermost and fully expanded leaves, and penultimate and fully expanded leaves corresponded to mature and early senescing leaves, respectively. The mRNA levels of *PHT4;4* were lowest in expanding leaves, maximal in the uppermost and fully expanded leaves, and slightly decreased in the penultimate and fully expanded leaves (Fig. 1a). The mRNA levels of *PHT2;1*, the other chloroplast envelope-localized PHT, showed similar trends (Fig. 1b). The mRNA levels of *TPT, ATPA*, *PETA*, *RBCL*, and *RBCS* were used as controls. For the Rubisco genes, their mRNA levels were highest in expanding leaves and rapidly decreased in lower leaf positions (Fig. 1f, g), as previously reported (Suzuki et al. 2009a). Similar trends were observed for the other control genes (Fig. 1c–e). Therefore, the uppermost and fully expanded leaves were used to examine the effects of RNAi suppression of *PHT4;4* on the mRNA levels.

**Fig. 2:**
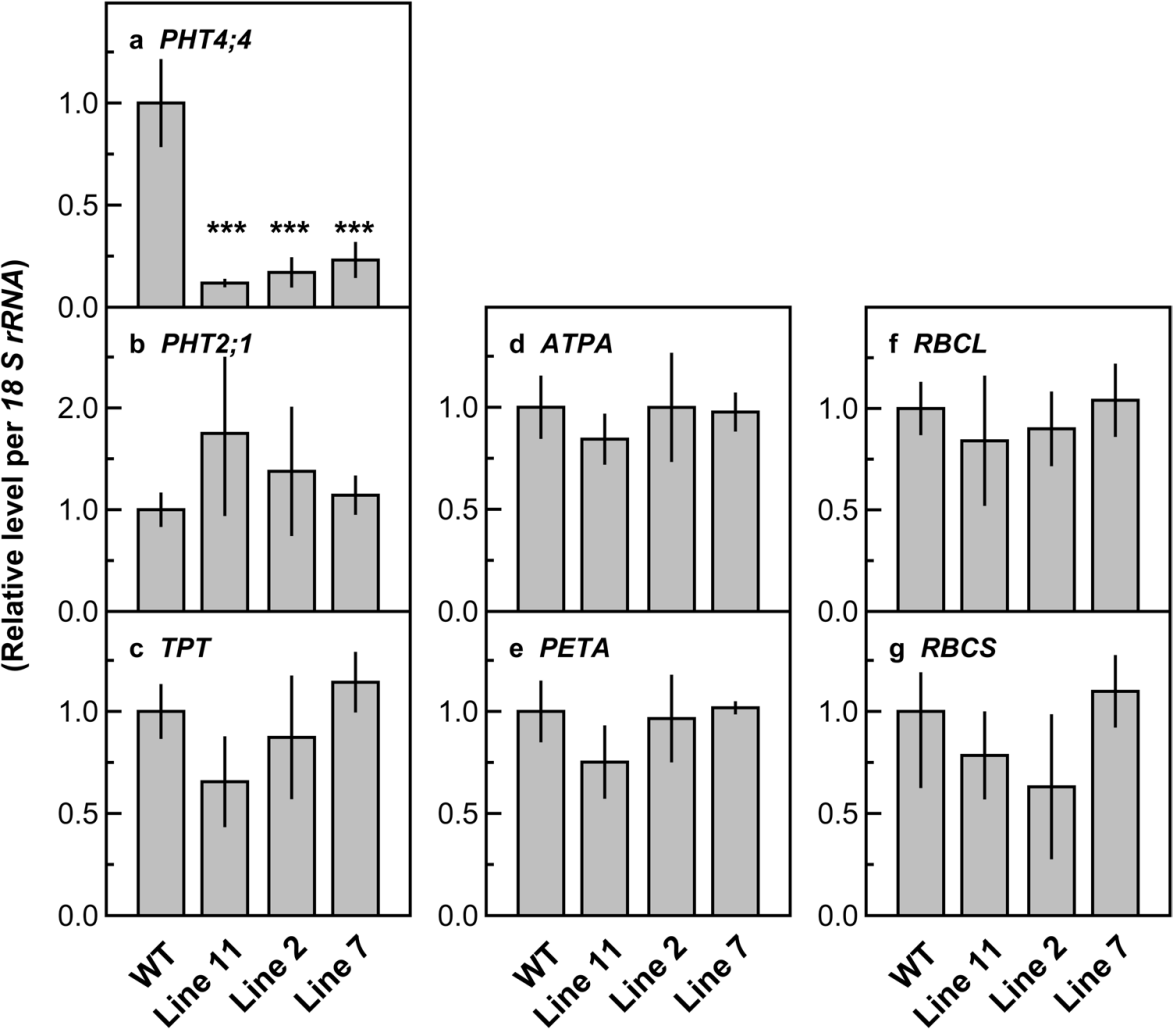
mRNA levels of the genes of phosphate transporters *PHT4;4* (**a**) and *PHT2;1* (**b**), triose phosphate/phosphate translocator (*TPT*) (**c**), the α subunit of chloroplast ATP synthase (*ATPA*) (**d**), the cytochrome *f* subunit of cytochrome *b*_6_/*f* complex (*PETA*) (**e**), and Rubisco genes *RBCL* (**f**) and *RBCS* (**g**) in the uppermost and fully expanded leaves of wild-type and transgenic plants with RNAi suppression of *PHT4;4* in rice. The mRNA levels of each gene were divided by the *l8 S rRNA* levels, which were measured in the same samples and then converted to relative values. The average value of mRNA level/*18 S rRNA* level in wild-type plants was defined as 1. Data are presented as means ± standard deviation (*n* = 3). Analysis of variance was performed on all leaves, followed by the Dunnett test using wild-type plants as controls. *** denotes a significant difference at *p* < 0.001

**Fig. 3:**
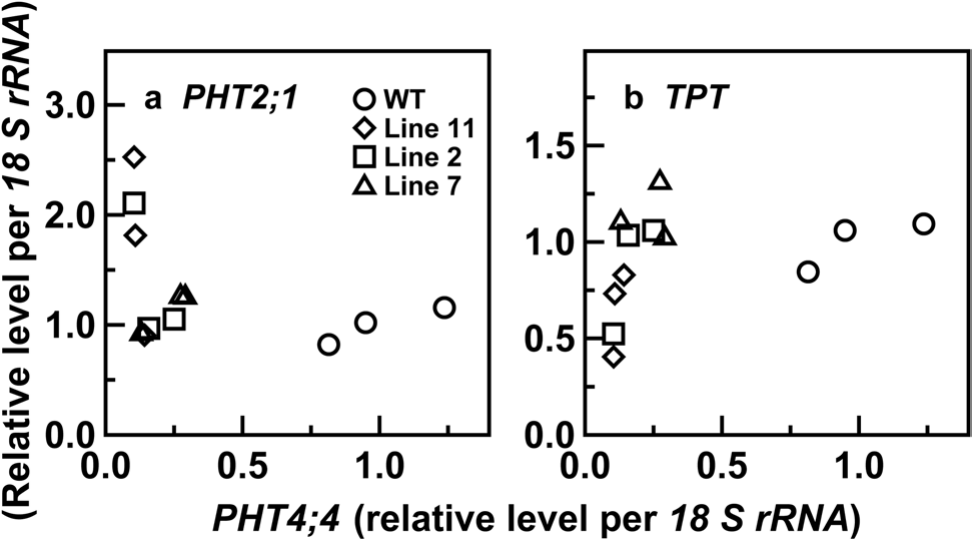
Relationships between mRNA levels of *PHT2;1* (**a**) or triose phosphate/phosphate translocator (*TPT*) (**b**) and those of *PHT4;4* in the uppermost and fully expanded leaves of wild-type and transgenic plants with RNAi suppression of *PHT4;4* in rice. Data are taken from Fig. 2. Circles, diamonds, squares, and triangles represent wild-type plants and lines 11, 2, and 8 of the transgenic plants, respectively. Spearman’s rank correlation coefficients in **a** and **b** are –0.427 and 0.595, respectively

In three lines of the transgenic plants (lines 11, 2, and 7), the mRNA levels of *PHT4;4* decreased to 12, 17, and 23% of those in the wild-type plants, respectively, indicating that gene expression of *PHT4;4* was substantially suppressed (Fig. 2a). The average mRNA levels of *PHT2;1* in the transgenic plants tended to be higher than that in wild-type plants, with the highest value being 175% in line 11 (Fig. 2b). The mRNA levels of *PHT2;1* tended to increase with decreasing mRNA levels of *PHT4;4*. In contrast, the average mRNA levels of *TPT* in lines 11 and 2 tended to be lower than that in the wild-type plants, with the lowest value being 66% in line 11 (Fig. 2c). The mRNA levels of *TPT* tended to decrease with decreasing mRNA levels of *PHT4;4*. Variations in the mRNA levels of *ATPA*, *PETA*, and *RBCL* among the genotype tended to be smaller than those of *PHT2;1* and *TPT* (Fig. 2d–f). The mRNA levels of *RBCS* in lines 11 and 2 tended to be lower than the other genotypes but were not necessarily correlated with those in *PHT4;4* (Fig. 2g). Thus, the mRNA levels of the photosynthetic genes examined were not notably affected with a certain trend in the transgenic plants. When the relationships between the mRNA levels of *PHT4,4*, *PHT2;1*, and *TPT* were examined, the mRNA levels of *PHT2;1* and *TPT* tended to increase and decrease, respectively, when those of *PHT4;4* extremely deceased (Fig. 3). Although we attempted to detect the PHT4;4 protein by immunoblotting using two different peptide antibodies we designed, these trials were unsuccessful (data not shown).

Immediately before beginning the gas exchange measurements followed by sampling, a notable growth inhibition was not observed in transgenic plants. Shoot length, tiller number, and root length did not substantially differ from those in the wild-type plants, although their tiller numbers tended to be slightly smaller, with the lowest value in line 11 (Fig. S1).

**Fig. 4:**
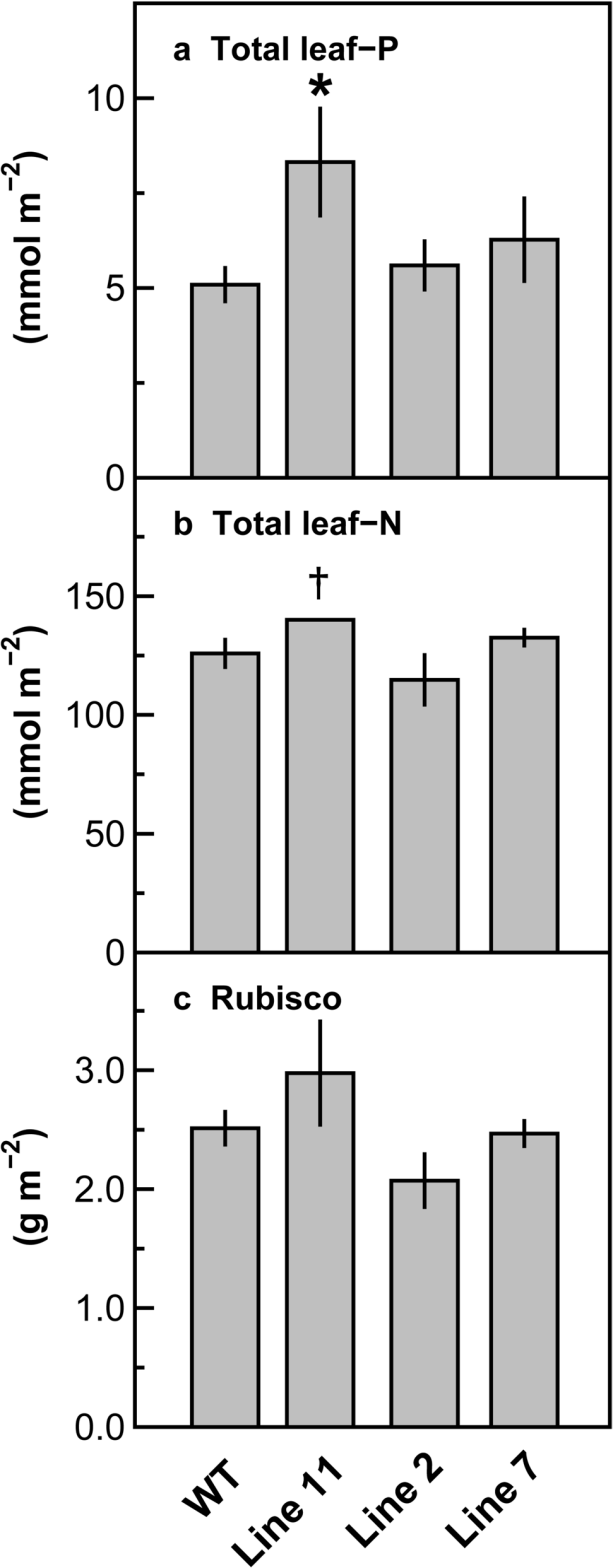
Total leaf-P (**a**) and leaf-N (**b**) levels and Rubisco levels (**c**) in the uppermost and fully expanded leaves of wild-type and transgenic plants with RNAi suppression of *PHT4;4* in rice. Data are presented as means ± standard deviation (*n* = 3). Analysis of variance was performed on all leaves, followed by the Dunnett test using wild-type plants as controls. ^†^ and * denote significant differences at *p* < 0.1 and *p* < 0.05, respectively

Total leaf-P levels in the uppermost and fully expanded leaves of the transgenic plants were not lower than those in the wild-type plants. The total leaf-P levels in lines 2 and 7 and wild-type plants were similar, whereas the total leaf-P levels in line 11 were 158% of those in the wild-type plants (Fig. 4a). Slight variations existed in the levels of total leaf-N and Rubisco, which is a large N sink in the leaves of C_3_ plants (Evans 1989; Makino et al. 1992) (Fig. 4b, c). Trends in the differences in total leaf-N levels among the genotypes were similar to those of Rubisco levels. In line 11, the total leaf-N and Rubisco levels tended to be higher than those in the other genotypes, as observed for the total leaf-P levels (Fig. 4a).

**Fig. 5:**
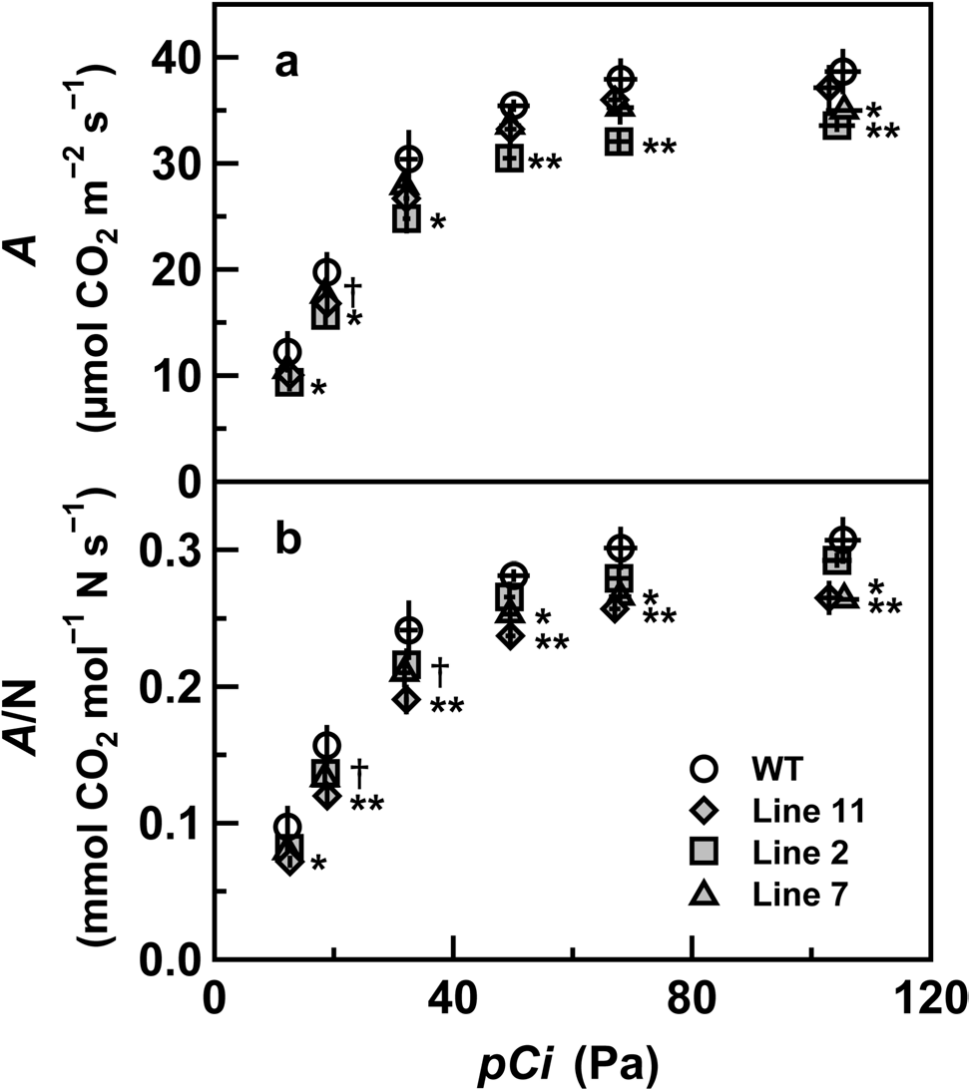
CO_2_ assimilation rates (*A*) in the uppermost and fully expanded leaves of wild-type and transgenic plants with RNAi suppression of *PHT4;4* in rice. Measurements were conducted under a photosynthetic photon flux density of 1500 µmol photons m^−2^ s^−1^, various intercellular CO_2_ partial pressures (*pCi*), leaf temperature of 25 °C, and a leaf-to-air vapor pressure difference of 1.0–1.2 kPa. Data are presented per unit leaf area (**a**) and per unit total leaf-N level (*A*/N) in **b**. White circles and gray diamonds, squares, and triangles denote wild-type plants and lines 11, 2, and 7 of the transgenic plants, respectively. Data are presented as means ± standard deviation (*n* = 3). Analysis of variance was performed on *A* in all leaves, followed by the Dunnett test using wild-type plants as controls. ^†^, *, and ** denote significant differences at *p* < 0.1, *p* < 0.05, and *p* < 0.01, respectively. When there are two symbols for the significant difference, lines 2 and 11 at *pCi* = 18.5 Pa and lines 2 and 7 at *pCi* = 105 Pa were significantly different in **a**, whereas lines 7 and 11 were significantly different throughout the range of *pCi* in **b**

*A* in the uppermost and fully expanded leaves of the transgenic and wild-type plants was measured under high irradiance and different CO_2_ levels (Fig. 5a). The values of *A* in the transgenic plants tended to be slightly lower than those of the wild-type plants under a wide range of CO_2_ levels, including *pCi* less than 20 Pa where *pCi*-response curves of *A* in rice are within the range of the initial slope (Makino et al. 1988, 1992, 1994; Suzuki et al. 2007, 2022c). *A* increased with increasing CO_2_ levels and was nearly saturated at approximately *pCi* = 60 Pa, irrespective of genotype. *A*/N was also calculated to evaluate the effects of *PHT4;4* suppression on CO_2_ assimilation (Fig. 5b). *A*/N values in lines 11 and 7 were slightly lower than those in wild-type plants by approximately 10–20% under different CO_2_ levels, whereas *A*/N values in line 2 tended to be marginally lower than those in wild-type plants. These decreases tended to be smaller than the decrease in *A* per unit of total protein level reported in previous studies (Häusler et al. 2000a; Yang et al. 2016).

## Discussion

### Gene expression of *PHT4;4* is most active in mature leaves

In the wild-type plants, the mRNA levels of *PHT4;4* were highest in the uppermost and fully expanded leaves among the leaves at different positions (Fig. 1a), indicating that gene expression of *PHT4;4* was most active in mature leaves during the course of leaf development. Similar trends were observed for *PHT2;1* (Fig. 1b). The half-lives of some PHT proteins are 1–12 h (Clarkson et al. 1992; Huang et al. 2013; Park et al. 2014). Owing to the short half-life of the PHT protein, gene expression of *PHT4;4* and *PHT2;1* is likely to be active when biochemical processes, including CO_2_ assimilation, are active. This expression pattern is different from that of Rubisco, which is active before leaf maturation to afford sufficient Rubisco in mature leaves (Loza-Tavera et al. 1990; Miller et al. 2000; Nikolau and Klessig 1987; Suzuki et al. 2001, 2010; Fig. 1f, g) owing to its long half-life of more than one week (Mae et al. 1983; Peterson et al. 1973; Simpson 1981).

### *PHT4;4* suppression does not decrease total leaf-P levels under P-sufficient conditions

The total leaf-P levels in the transgenic plants were not lower than those in the wild-type plants despite the substantially decreased mRNA levels of *PHT4;4* (Figs. 2a, 4a), suggesting that PHT4;4 does not notably contribute to P_i_ supply to leaves under P-sufficient conditions. Similar trends were observed in the rice mutants defect of PHT2;1, whereas total leaf-P levels decreased to some extent under P-deficient conditions (Liu et al. 2020). Thus, chloroplast envelope-localized PHTs are likely to play a minor role in P_i_ supply to leaves under P-sufficient conditions. Total leaf-P levels increased in line 11 (Fig. 4a), but its causes remain unclear. *PHT2;1* gene expression in line 11 tended to be upregulated as mentioned later (Figs. 2b, 3a). However, this upregulation of *PHT2;1* is unlikely to be primarily responsible for the increased total leaf-P level due to its limited role in P supply to leaves as suspected above. In addition, the differences in total leaf-P level among the genotypes did not show the pattern similar to those in the mRNA level of *PHT2;1* (Figs. 2b, 4a). In line 11, total leaf-N and Rubisco levels also tended to increase (Fig. 4b, c), while plant growth was relatively inferior to the other genotypes due to the decreased tiller number (Fig. S1). It cannot be denied that P and N were concentrated in the tissues due to the relatively inferior plant growth, although its causes remain unclear.

### Substantial suppression of *PHT4;4* causes slight symptoms of P_i_-limited CO_2_ assimilation

A typical symptom of P_i_-limited CO_2_ assimilation is enhanced CO_2_ insensitivity of *A*, which is observed as unchanged or even decreased *A* with increasing CO_2_ levels (Busch and Sage 2017; Fabre et al. 2019; Sage and Sharkey 1987; Sharkey 1985b; Yang et al. 2016). When substantial *TPT* suppression restricted P_i_ import to chloroplasts, *A* per unit total protein level under low-to-normal CO_2_ levels decreased by more than 20% (Häusler et al. 2000a; Yang et al. 2016). In the transgenic rice plants with *PHT4;4* suppression, enhanced CO_2_ insensitivity was not observed according to the shapes of the *pCi*-response curves of *A*, whereas *A*/N slightly decreased (Fig. 5). These results indicate that slight symptoms of P_i_-limited CO_2_ assimilation were observed in the transgenic plants.

In the transgenic plants, gene expression of *PHT2;1* or *TPT*, which could be involved in the maintenance of chloroplastic P_i_ homeostasis, tended to be affected. The mRNA level of *PHT2;1* and *TPT* tended to increase and decrease, respectively, when the mRNA level of *PHT4;4* extremely decreased (Figs. 2b, c, 3). These results suggest that gene expression of *PHT2;1* and *TPT* was upregulated and downregulated, respectively, in response to extreme *PHT4;4* suppression. These changes in gene expression might affect CO_2_ assimilation in the transgenic plants. However, the magnitude of the decreases in *A*/N did not substantially differ among the transgenic lines, including line 7 with the mRNA levels of *PHT2;1* and *TPT* scarcely altered (Figs. 2b, c, 5b). Therefore, substantial suppression of *PHT4;4* was primarily responsible for the slight symptoms of P_i_-limited CO_2_ assimilation. It is suggested that PHT4;4 is involved in the maintenance of chloroplastic P_i_ homeostasis for healthy CO_2_ assimilation, but that its control on CO_2_ assimilation is limited. The slight symptoms of P_i_-limited CO_2_ assimilation were also observed in line 11 despite the increased total leaf-P level (Fig. 4a), implying that the increased P in leaves was sequestered in vacuoles, which accumulate approximately 85–95% of total leaf-P as a non-metabolic P pool under P-sufficient conditions (Hawkesford et al. 2012; Lauer et al. 1989).

The small control of *PHT4;4* on CO_2_ assimilation would be explained from the magnitude of its contribution to P_i_ import to chloroplast. Defect of PHT4;4 in Arabidopsis scarcely affected *in-vivo* stromal P_i_ concentration measured using a FRET-based system as mentioned before (Raju et al. 2024), whereas TPT notably contributes to P_i_ import to chloroplasts (Riesmeier et al. 1993). Therefore, P_i_ transport capacity of PHT4;4 during photosynthesis is likely to be lower than that of TPT, leading to the small effects of its suppression on CO_2_ assimilation.

Disturbed chloroplastic P_i_ homeostasis is reported to be accompanied by changes in leaf carbohydrate status. Accumulation of starch was observed in transgenic potato plants and Arabidopsis mutants with suppression or defect of TPT (Riesmeier et al. 1993; Schneider et al. 2002; Walters et al. 2004). In contrast, leaf starch levels decreased in rice mutants defect of TPT or PHT2;1, another chloroplast envelope-localized PHT (Lee et al. 2014; Liu et al. 2020). Effects of *PHT4;4* suppression on leaf carbohydrate status would provide further information on the role of PHT4;4 in chloroplastic P_i_ homeostasis.

### Upregulated *PHT2;1* gene expression does not fully resolve the P_i_-limited CO_2_ assimilation caused by substantial *PHT4;4* suppression

The upregulated *PHT2;1* gene expression can be interpreted as a compensation for extreme *PHT4;4* suppression (Figs. 2b, 3a), implying the cooperation of these PHTs in the maintenance of chloroplastic P_i_ homeostasis for CO_2_ assimilation. In contrast, the downregulated *TPT* gene expression would have negative impacts on this function (Figs. 2c, 3b), although its physiological significance is difficult to speculate at present. Slight decreases in TPT activity in *TPT*-antisense transgenic tobacco plants led to slightly decreased *A* under strong light intensities, normal or saturated CO_2_ levels, and low O_2_ levels (Häusler et al. 2000b). In the present study, it was difficult to distinguish the effects of *PHT4;4* suppression from those of *TPT* downregulation on *A* and *A*/N (Fig. 5). At least, the upregulated *PHT2;1* gene expression did not fully resolve the negative effects of *PHT4;4* suppression and concomitant *TPT* downregulation, if any, implying that PHT2;1 plays a minor role in the maintenance of chloroplastic P_i_ homeostasis for CO_2_ assimilation as in PHT4;4. In contrast, *PHT2;1* overexpression has been suggested to notably affect P use of chloroplasts. In *PHT2;1*-overexpressing rice plants with its mRNA levels increased to approximately sixfold, total P content of isolated chloroplasts increased to more than 1.5-fold (Bouain et al. 2022). Effects of *PHT2;1* overexpression on CO_2_ assimilation remain to be examined.

## Conclusion

In the present study, we suggest that PHT4;4 is responsible for chloroplast P_i_ homeostasis for healthy CO_2_ assimilation to a limited extent. P_i_ limitation can hinder photosynthesis improvement. In transgenic rice plants overexpressing Rubisco (Makino and Sage 2007; Suzuki et al. 2007, 2009a), increases in *A* are in a limited extent under low-to-normal CO_2_ levels and high irradiance, while Rubisco is assumed to determine *A* (Farquhar et al. 1980; von Caemmerer 2000). Simultaneously, *A*/N tend to be slightly lower than that in the wild-type plants. The CO_2_ insensitivity of *A* is slightly enhanced at elevated CO_2_ levels. Decreases in the *in-vivo* Rubisco activation state, which is a symptom of P_i_-limited CO_2_ assimilation (Sage et al. 1988; Sharkey et al. 1986a, b), also occur. To solve these problems of P_i_-limited CO_2_ assimilation for improving photosynthesis, means other than the use of PHT4;4 need to be explored. Conversely, it was shown that *PHT4;3* overexpression in Arabidopsis and *PHT2;1* overexpression in rice led to notably increased P content of chloroplasts, accumulation of phytic acid, and inhibited plant growth under elevated CO_2_ levels and low light intensities (Bouain et al. 2022). Such subtle nature of plant P use need to be paid attention to.

## Supplementary Information

Below is the link to the electronic supplementary material.

Supplementary file1 (PDF 26 KB)
