# Supplementary material for: Effects of suppression of phosphate transporter 4;4 on CO2 assimilation in rice
Source: J Plant Res. 2025 Apr 18;138(4):667–77. doi: 10.1007/s10265-025-01638-4 (PMC12238151; doi:10.1007/s10265-025-01638-4)
Supplement: Supplementary file 1 — Supplementary file1 (PDF 26 KB) [file 10265_2025_1638_MOESM1_ESM.pdf]

Electronic supplementary materials

Title:

Effects of suppression of phosphate transporter 4;4 on CO_2 assimilation in rice

Authors:

Ryosei Harada[1], Takaya Sugimoto[2], Yuki Takegahara-Tamakawa[2], Amane Makino[3,4], Yuji Suzuki[2]

[1]Graduate School of Arts and Sciences, Iwate University, Morioka, Japan; [2]Faculty of Agriculture, Iwate University, Morioka, Japan; [3]Graduate School of Agricultural Science, Tohoku University, Sendai, Japan; [4]Present address: Institute for Excellence in Higher Education, Tohoku University, Sendai, Japan.

Journal:

Journal of Plant Research

Corresponding author:

Yuji Suzuki (Faculty of Agriculture, Iwate University, 3-18-8 Ueda, Morioka 020-8550, Japan)

Tel and Fax: +81-19-621-6153

E-mail: ysuzuki@iwate-u.ac.jp

Content:

Fig. S1

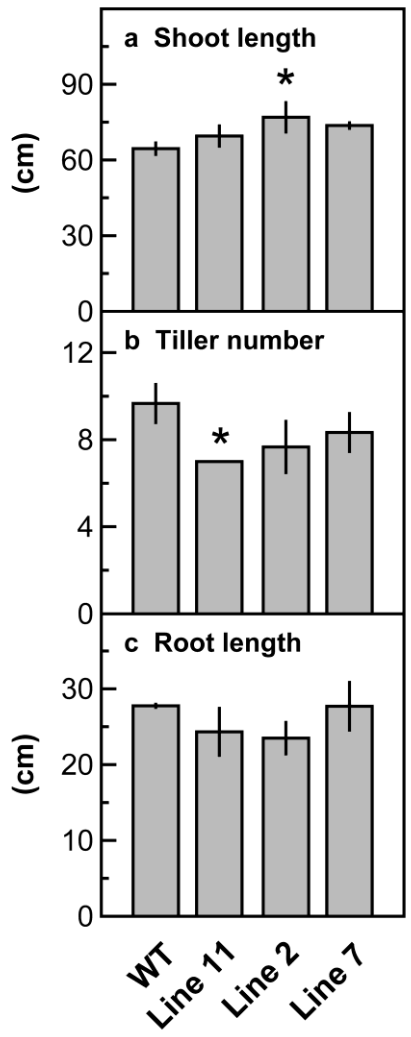

Fig. S1 Shoot length (**a**), tiller number (**b**), and root length (**c**) in wild-type and transgenic plants with RNAi suppression of *PHT4;4* in rice immediately before the beginning of CO_2 gas exchange measurements. Data are presented as means ± standard deviation (n = 3). Analysis of variance was performed, followed by the Dunnett test using wild-type plants as controls. * denotes a significant difference at $p < 0.05$.
